# The ‘STRICT’ framework for promoting effective malaria control in Ghana

**DOI:** 10.1186/s12936-024-05146-z

**Published:** 2024-11-11

**Authors:** Irene G. Ampomah, Susan Devine, Genevieve A. Ampomah, Theophilus I. Emeto

**Affiliations:** 1 Department of Population and Health, University of Cape Coast, UC 182, Cape Coast, Ghana; 2 Public Health and Tropical Medicine, James Cook University, Townsville, QLD 4811 Australia; 3 Department of Sociology, Kwame Nkrumah University of Science and Technology, Kumasi, Ghana; 4 World Health Organization Collaborating Center for Vector-Borne and Neglected Tropical Diseases, James Cook University, Townsville, QLD 4811 Australia

**Keywords:** Effective, Framework, Healthcare providers, Herbal medicine, Integrated healthcare, Integration, Malaria, Model, Traditional herbal medicine

## Abstract

**Background:**

Malaria remains a significant public health burden, necessitating evidence-based strategies to reduce prevalence and associated morbidity. This study explores the potential of integrated healthcare, encompassing both modern and traditional herbal medicine (THM), for malaria control in Ghana.

**Methods:**

Employing a qualitative approach, semi-structured interviews were conducted with medical doctors, pharmacists, and THM providers. Thematic analysis approach was utilized to inductively analyse interview data and integrate participants’ lived experiences and suggestions.

**Results:**

Six themes emerged: Standardization of THM practice; Training on THM broadened; Research on THM expanded; Increasing awareness of THM integration hospitals and inclusion of THM in national health insurance scheme; Constant supply of certified herbal medications; and Tax relief provision. These recommendations form the ‘STRICT’ framework for developing functional health system for promoting an effective malaria control through integrated healthcare in Ghana.

**Conclusion:**

It was evident that the ‘STRICT’ framework can potentially transform healthcare delivery and improve service quality for malaria patients. Policymakers, healthcare providers, and managers can utilize these insights to advocate for and implement integrated healthcare strategies, ultimately enhancing service delivery for all Ghanaians, particularly those suffering from malaria.

## Background

Malaria remains a significant public health burden in Ghana, with an estimated 40% of all outpatient visits attributed to the disease [1]. Children under five years and expectant mothers are particularly vulnerable [2, 3]. Nationwide surveys report a high prevalence of malaria parasites, averaging around 28% among children aged 6–59 months [4, 5].

Transmission patterns vary across Ghana’s diverse ecological zones. The savannah belt experiences a single peak in malaria prevalence during the wet season (June to October) [6–10]. In contrast, coastal and forest zones experience two annual peaks [7, 10, 11].

## Malaria interventions in Ghana

Ghana has a long history of deploying malaria control strategies, primarily focused on eliminating either the parasite within the host or the *Anopheles* mosquito vector [3]. Vector control interventions like indoor residual spraying and insecticide-treated bed nets [ITNs] have been utilized since the colonial era, initially concentrated in major cities [12]. Nationwide ITN distribution commenced in 2004, followed by a policy subsidizing their delivery in 2007 [3]. Indoor residual spraying has also been implemented, albeit on a limited scale since 2005. Despite these limitations, research suggests high coverage levels for both ITNs and artemisinin-based combination therapy (ACT) in Ghana [12].

Pre-independence interventions targeted the parasite through various medications including amodiaquine-pyrimethamine, daraprim or pyrimethamine, primaquine, lapudrine and chloroquine [3]. From 1950 to 2000, single therapies such as chloroquine and quinine dominated treatment protocols. However, widespread parasite resistance to *chloroquine* necessitated a policy shift in 2004. Artesunate-amodiaquine replaced chloroquine as the first-line treatment for uncomplicated malaria. Subsequent revisions in 2007 and 2009 incorporated artemether-lumefantrine and dihydroartemisinin-piperaquine into the treatment regime [13]. The high cost of these modern artemisinin-based combinations poses a challenge for many Ghanaians [14, 15]. This has fuelled interest in exploring the potential of traditional medicine (TM) for malaria treatment, with its perceived benefits of lower costs and fewer side effects [14].

### The potential of integrated healthcare

TM, defined by the World Health Organization (WHO) as the “sum total of knowledge and practices used in diagnosis, prevention and elimination of physical, mental and social imbalance” [16, 17], often utilizes plants for medicinal purposes [18]. In this study, TM refers to the use of medicinal plants (traditional herbal medicine [THM]) for treatment.

While studies report high prevalence of THM use [19], and coverage levels of ACT and ITNs [12] in Ghana, malaria burden remains significant [1]. This highlights the limitations of current approaches and underscores the potential value of a multidisciplinary or integrated healthcare approach to achieve the national goal of malaria eradication by 2030 [12].

An integrated healthcare approach in Ghana involves the formal incorporation THM into the mainstream health system [20]. This inclusive approach [21, 22], is evident through the establishment of THM policies, the integration of THM clinics into government hospitals, the creation of research centers like the Center for Scientific Research into Plant Medicine (CSRPM), and the incorporation of THM into tertiary education [22–24]. These initiatives signify a recognition of THM as a legitimate medical practice within the Ghanaian healthcare landscape. Consequently, orthodox and THM providers are expected to collaborate harmoniously, fostering mutual trust and respect to provide comprehensive and well-coordinated services to the population.

However, previous research suggests a non-functional integrated healthcare system in Ghana, advocating for evidence-based collaboration between healthcare providers and service users [24–28]. Notably, research exploring malaria control through integrated healthcare practices is scarce. Additionally, there’s limited understanding of how key stakeholders, such as medical doctors, pharmacists, and THM providers, perceive and envision an effective integrated approach for reducing malaria prevalence in the Ghanaian context.

Therefore, the current study intends to confirm evidence and develop a framework to promote effective management of malaria through the integration intervention. The study is guided by the research question: how can malaria control be improved through the practice of integrated healthcare in Ghana? Insights gained from study participants through the pluralist approach (merging participants’ views to achieve a mutual understanding on the apposite approach for the development of functional health system) could inform the modification of policies and activities that might promote the delivery of quality healthcare, boost the efficiency of the integration programme and lead to lower disease prevalence, especially malaria cases [29].

## Methods

### Research design

The study employed a qualitative design grounded in the principles of the pluralistic evaluation approach [29] and guided by the Consolidated Criteria for Reporting Qualitative Studies (COREQ) [30]. This approach explored the experiences and recommendations of key stakeholder groups (healthcare practitioners) within the Ghanaian health system regarding integrated healthcare and malaria control. Thus, the research sought to empirically explore the perspectives and experiences of orthodox healthcare providers (medical doctors and pharmacists) and THM practitioners regarding strategies to effectively mitigate malaria prevalence and associated morbidity and mortality within an integrated healthcare framework.

### Study participants

Participants included medical doctors (MD), pharmacists (PM) and THM providers practicing in three ecological zones of Ghana: the forest zone (Kumasi metropolis), the coastal zone (Cape Coast metropolis), and the savannah zone (Wa municipality). Participants were eligible for inclusion if they were aged 18 years or above and were working within these designated study settings during the research period. Purposive sampling, a non-probabilistic sampling technique, was employed to recruit participants to ensure the inclusion of individuals who possess specific characteristics or expertise relevant to the research study [31]. This method involved: (1) Identification of eligible participants through preliminary scouting or a pre-data collection survey; (2) Selection and contact of participants who could provide relevant information for the interview questions; and (3) Obtaining informed consent and commitment to participate in the research.

### Data collection

To gain insight into healthcare providers’ experiences with integrated healthcare practices and malaria control, semi-structured, individual, face-to-face in-depth interviews were conducted. These interviews lasted approximately 50 min each and were audio recorded with the permission of participants. The semi-structured interview approach allowed the researcher to prepare questions beforehand to guide the discussion while permitting in-depth exploration of the research topic [32]. The interview guide comprised seven key questions developed based on Donabedian’s framework for evaluating healthcare quality [33] and covered topics centered on participants’ views and experiences relating to malaria management through THM integration in Ghana. Specifically, the discussions focused on ways to effectively treat/reduce malaria cases through the practice of integrated healthcare. The guide was pre-tested on six healthcare providers (two each from MD, PM, and THM categories).

The pre-test interviews were conducted by the principal investigator (IGA) and reviewed by TIE to assess the appropriateness and reliability of the questions. Feedback from the pre-test interviews was used to refine the final interview guide.

Data collection spanned from July to October 2023. Three research assistants (two males and one female) with master’s degrees in public health and experience in qualitative research were recruited from the University of Development Studies (UDS) and University of Cape Coast (UCC) to help with data collection. The study objective and data collection instrument (interview guide) were explained to the assistants at a training session that took place in the fourth week of June 2023 at UCC campus. Every session lasted for four hours. The training enhanced the assistants’ understanding of the study.

Each interview began with obtaining informed consent from the participant and concluded with a brief review of the interview content to ensure accuracy and mutual understanding. Key topics of interest included participants’ experiences with integrated healthcare in malaria control, as well as their recommendations for effective management to reduce malaria prevalence. Probing techniques were used to elicit detailed responses. Data collection ceased upon reaching data saturation, a point at which no new information emerged to enhance the researchers’ understanding of healthcare integration and quality malaria control [34]. The research assistants documented their observations during the data collection exercise. The first-named author (IGA) observed the first five interviews to ensure accuracy and homogeneity in the interviewing method, however no discussion occurred between the interviewees and IGA.

### Data analysis

Audio-recorded interviews were transcribed by a professional transcriber. All identifiable information was removed, and participant identifiers were assigned. The transcribed data were then imported into NVivo software version 12 (QSR International Pty Ltd., Victoria, Australia). The data analysis process involved five stages: (1) familiarization with the transcribed data to identify codes. At this stage the authors read the transcripts thoroughly and familiarize ourselves with the data by noting salient points; (2) next, the codes were grouped into themes based on their similarities (for example, codes that related directly to service delivery were classified under the broad term ‘practice’); (3) classifying of themes into thematic networks based on their conceptual content (issues relating to policies/scientific practices were grouped under standardization of THM practice); (4) additional exploration of thematic networks for cause-and-effect associations. After grouping the themes, further assessment showed direct relationships between the thematic networks, where themes such as standardization of THM practice and expanding training/research on THM had the tendency to improve collaborations between the healthcare providers; whereas, provision of tax relief could promote financial security; (5) developing a model/framework connecting the conceptual findings in the thematic network to the research question (synthesis of the six main themes led to the development of the ‘STRICT’ framework, which answered the research question). Data coding and generation of themes were performed independently by IGA and TIE and confirmed by GAA. Disagreements regarding the coding and emerged themes were resolved in a consensus meeting.

## Results

Forty-five (45) participants comprising 19 THM practitioners, 15 pharmacists and 11 medical doctors took part in the study. Most of the participants originated from the coastal (Cape Coast) 36.6% and forest belts (Kumasi) 36.6%, while 26.8% were recruited from the savannah belt (Wa). The majority of the study participants were males accounting for 65.9%. Participants’ average ages were 31 years for medical doctors, 36 years for pharmacists and 35 years for THM providers. All except 10 participants (THM providers) worked in private facilities. Three of the medical doctors mentioned pediatric, emergency, and internal medicines as areas of specialization. Two pharmacists identified as pharmacy technicians, while one of the pharmacists from the Kumasi metropolis reported being an expert in antimicrobial resistance.

### Participants’ perspectives on promoting effective malaria control through the practice of integration

**Fig. 1:**
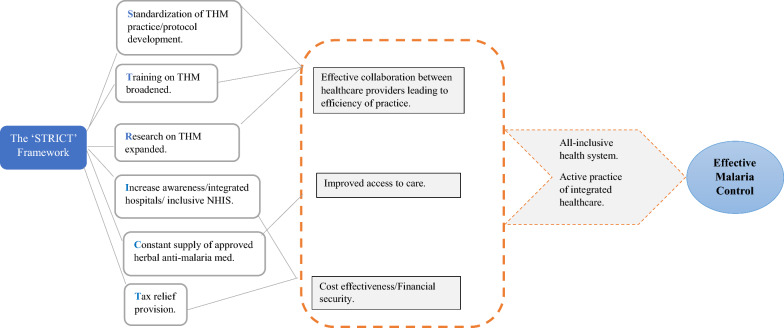
The ‘STRICT’ framework for promoting effective malaria control in Ghana.

Six themes emerged from the data. **S**tandardization of THM practice, Training on THM broadened, Research on THM expanded, Increase awareness/integrated hospitals/inclusive National Health Insurance Scheme NHIS, Constant supply of approved herbal anti-malarial medications and Tax relief provision. Based on these themes, the ‘STRICT’ framework (Fig. 1) is presented for promoting and sustaining effective malaria control through the practice of integrated healthcare in Ghana.

### Standardization of THM, strict policy implementation and integration protocol development

Medical doctors and pharmacists perceived that standardizing THM practice through rigorous implementation of policies governing the field and the development of an integration protocol could foster effective malaria control. The medical doctors further explained that the ministry of health must enact a policy to ensure THM centers are equipped with the needed resources to deliver their services efficiently, especially to malaria patients and the THM providers should adopt a more scientific means of dosage to meet the standards of orthodox medicine. The pharmacists also felt that the government should design an integration protocol that could bring all stakeholders on board and ensure their full participation in the integration intervention.

*The Ministry of Health should make sure that the policies they bring involve the medical practitioners and the medical staff. The policies must ensure that the herbal units have the resources and medications to provide malaria treatment*,* and such policies should be implemented well* [P 34, MD, Cape Coast].

*For the orthodox medicine*,* we have the graduated scale. So*,* when you buy an orthodox syrup*,* you will see that it comes with this plastic cup that the millimetres written on it. So*,* if they [TM practitioners] can do same*,* then it will be better than this spoon thing. So*,* it will take away the challenge with dosage* [P 33, MD, Wa].

*I don’t think there has been enough stakeholder engagement to bring all the key stakeholders on board. If it has been done*,* then I don’t think it was properly done. That is the major challenge. The government should design an integration protocol to bring all the stakeholders on board* [P 27, PM, Cape Coast].

### Training on THM broadened

Participants reported that training on THM was not adequate, and this has led to limited or no knowledge on THM among orthodox medical providers, hence medical doctors discouraging patients from patronizing THM even at approved centres. Therefore, all providers irrespective of the field of medicine suggested that THM training should be broadened by increasing the number of THM training institutions, including THM in medical school curriculum and joint training for both providers. This they believe would help identify the limitations of each medical field, eliminate rivalry between the healthcare providers and promote effective collaboration in the health system.

*…we don’t have enough specialised institutions where we can go for training. It is like the government has not created institution or structure for us to receive training. Because of that*,* we don’t have much training. So*,* I think the government should invest more in creating institutions to train us the traditional practitioners* [P 13, THM provider, Wa].

*I think we should incorporate herbal medicine in the general training of medical doctors because most medical doctors have little to no information on TM. And so*,* we tend to discourage our patients just because we are not aware of how it should be given. So*,* it should be incorporated into the curriculum of medical doctors so that you will be aware of the services available*,* what they are capable of doing*,* so that we can work together* [P 39, MD, Kumasi].

*…the practitioners of both areas*,* those with orthodox and those in herbal should have a joint training or education so that they will see the limitation of each practice*,* how they should relate so that*,* it does not appear as though the two parties are competing but coming together to solve the health problem* [P 22, PM, Wa].

### Research on THM expanded

The participants from the forest and coastal belts explained that children present with the most severe forms of malaria; however, most THM cannot be prescribed for people aged 12 years and below due to insufficient research supporting the administration of herbal medicine to this age group. Participants proposed that more research centers should be established and studies on THM must be intensified to eliminate any trial and error in combining it with conventional therapies for treating malaria. They also believed that increased research could lead to the manufacturing of suitable herbal anti-malarial medications for adult and children, thereby offering options in the care process.

*We must also research more into TM for malaria control to be very sure that we are not doing trial and error. So*,* the government should establish more research centers to help in that regard* [P 32, MD, Cape Coast].

*Malaria is a big problem*,* but the herbal medicine is such that it is not allowed to be given to children under 12 years. They think that there are not enough studies to support the administration of the medicine to children. Meanwhile*,* malaria affects children more than adults. So*,* we need more studies on how these herbal medicines work*,* and whether they can be combined with orthodox medicines*,* and how effective it will be in treating malaria in children. Children present the worst form of malaria. And so*,* if we have alternative for them*,* then it will help* [P 29, PM, Kumasi].

*There are other herbal medicines on the market which have proven to be effective in treating malaria. But we only source our herbal medicines from one place*,* that is*,* the Centre for Plant Medicine Research*,* we cannot prescribe medications outside of that. So*,* if we run out of that medicine*,* then it means we are handicapped. So*,* there must be more research centres and research activities on herbal medicines for treating malaria so that we can have alternatives; because when you go to the orthodox side*,* there are malaria medications from brands. So*,* if herbal medicine field does that*,* it would help* [P 14, THM provider, Kumasi].

### Increase awareness, integrated hospitals and NHIS inclusiveness

Most pharmacists in the forest belt recommended increasing community awareness of the integration of traditional approaches to malaria treatment and THM. They believed that the Ghana Health Service must sensitize people to be aware of the presence of THM clinics in government hospitals, where scientifically proven herbal anti-malarial medications are available and encourage the public to patronize THM services/products from such facilities. The participants also felt that one useful way to sensitize community members/service users is by developing a jingle that could be played at the premises of the health facilities. They further suggested that the various radio stations and information centers could be used as communication channels to disseminate information on integrated health services and malaria treatment.

*The Ghana Health Service should let the community know that if they need herbal medicines for malaria there are proven medicines in the hospital. That will encourage them to access it. The radio and information centers should broadcast it. The hospital should get a jingle that will be broadcasted at the hospital premises to create awareness that there are herbal medicines for treating malaria* [P 29, PM, Kumasi].

*… education through radio and TV can help. More of this education should be done in the south (forest and coastal zones). Because over here*,* you can say that the integration is going well. So*,* they should implement the same thing in the south to accommodate them (medical herbalists)* [P 43, PM, Wa].

*… We need a lot of publicity and education*,* especially to the public for them to know that there is a qualified medical herbalist who can give them the best service* [P 18, THM provider, Kumasi].

*… the solution is education just as I mentioned with the use of megaphones to make people aware of the THM unit in the facility. We should start using our megaphones to educate people. We can also go to the radio stations to raise awareness. Because*,* when you educate people*,* they get to know*,* and understand*,* which make them patronize the services* [P 37, MD, Kumasi].

In addition, the notion that malaria could be effectively managed by increasing integrated hospital and making the NHIS inclusive were notable among participants in the savannah belt. They narrated that service users already have a perception that THM are effective in curing ailments, hence, they will go to every length to secure such medications. Therefore, to make such health services and products geographically and financially available, the government should increase the number of integrated hospitals and make the national health insurance more comprehensive by including approved THM in the NHIS. Participants believed that covering or subsidising the cost of certified herbal anti-malarial medications through the national health insurance could motivate malaria patients to utilize services and medications at THM clinics in integrated health centers.

*People already have the perception that the herbals are good*,* or because it is made of plant*,* it is good. Once they have that mentality*,* they will go every distance to get the herbal medicine other than the orthodox drug. So*,* incorporating it well into the hospital setting and increasing the number of the hospitals with the herbal clinics will help* [P 25, PM, Wa].

*…just as the orthodox medicines have been placed on the National Health Insurance List*,* they should have some traditional herbal medicines probably for malaria for a start*,* to be covered by insurance. Because if I have my health insurance card and my previous treatment of malaria*,* I received the orthodox medicine free of charge with the help of the insurance*,* I don’t see why in that same facility*,* I will go to pay for traditional medicine to treat malaria. I should be motivated enough to move from using orthodox medicine to using THM* [P 22, PM, Wa].

*The other thing is about the cost-effectiveness of THM. I am not sure whether it is cash-and-carry*,* but it looks like they pay. So*,* I think probably*,* the government can consider putting it on the national health insurance* [P 45, MD, Wa].

### Constant supply of approved herbal anti-malaria medications

Participants reported that while the practice of integrated healthcare is good, they felt the facilities are not well stocked. Therefore, they proposed that the government must ensure constant supply of approved THM, especially herbal anti-malarial medications. They explained that this could be achieved by establishing certified, functional herbal research and production centers to ensure constant supply of FDA certified medications.

*The integration is good. It is excellent but let’s stock them. Assuming we have about ten well-certified and qualified herbal production and research centers supplying Ministry of Health or Ghana Health Service with herbal medications that have been approved by the FDA on a constant basis*,* I don’t think we will go and buy artemether. Government must ensure constant supply of the approved herbal anti-malaria medications* [P 34, MD, Cape Coast].

### Tax relief provision

One interesting proposal from the study participants has to do with the government granting tax relief to THM providers/manufacturers. This recommendation was notable among the THM providers who operated in private health facilities. They recounted that they pay a lot of taxes, which is causing a financial burden on them. Therefore, they advised that the ministry of health together with the central government should offer tax relief to ease the economic burden, which they believe would lead to lower prices of certified THM.

*When I take our herbal clinic*,* there are so many taxes that we pay. That becomes a burden and affects our output. So*,* at least the Ministry of health in collaboration with the government can subsidize or provide tax relief for the producers of TM malaria treatments so that the cost of the TM will be lower* [P 5, THM provider, Cape Coast].

## Discussion

This qualitative study explored the perspectives of key stakeholders within the Ghanaian health system including medical doctors, pharmacists and THM providers regarding the promotion of effective malaria control through integrated healthcare practices. The research aimed to develop a framework for improved malaria management in Ghana. The study findings revealed that standardization, training, research, and sensitization initiatives focused on THM are crucial for delivering quality malaria care. Participants emphasized the need for policy modifications to facilitate financial support from the government, enhance user engagement, and foster interprofessional collaboration among healthcare providers. Similar findings have been reported by earlier studies where effective integration and access to trained/experienced healthcare providers yielded better health outcomes [35–37].

### Strengthening THM integration

The elimination of unqualified THM providers is key to an effective integrated health system and achieving a reduction in malaria prevalence [24]. This highlights the importance of regulating and standardizing the THM field. However, participants expressed concerns regarding the perceived inadequacy of existing efforts, such as the Traditional Medicine Practice Act—2000 (Act 575), and associated regulatory bodies [24, 25, 38, 39]. Uncertainties regarding current THM regulations and a perceived lack of effectiveness in ensuring a consistent supply of certified herbal antimalarial medications were identified as key issues. Participants advocated for stricter implementation of regulations, standardized dosages, and the development of integrated treatment protocols, potentially leading to increased user confidence in THM [39]. This, in turn, could facilitate collaboration between orthodox medicine providers and encourage a more effective referral system [25, 26, 40], , ultimately contributing to reduced malaria cases.

### Enhancing training and research

The emphasis on THM training and research by participants suggests a perceived need for a robust foundation in effective malaria control and integrated healthcare practices. This potentially reflects a gap in current educational programs for biomedical healthcare providers regarding herbal medicines. The study findings support incorporating THM education into the curriculum of medical doctors and pharmacists, alongside expanding the number of THM training and research institutions. This could equip healthcare providers with a deeper understanding of herbal anti-malarial medications, potentially leading to improved service delivery and a more consistent supply of certified herbal treatments. Policies incentivizing healthcare providers to acquire knowledge in THM could be crucial for enhancing the quality of care and the effectiveness of the integrated healthcare system in Ghana [25, 38, 41–43].

### User sensitization and financial considerations

Participants identified sensitization as a key factor in promoting the use of integrated healthcare services and optimizing malaria control efforts. Ongoing communication strategies highlighting the availability of integrated hospitals and certified herbal anti-malarial medications were perceived as crucial for facilitating access to quality care. Continuous information dissemination could also encourage medical doctors and other biomedical practitioners to embrace integrated practices and refer patients when appropriate. Consequently, mass public awareness campaigns utilizing print, visual, and audiovisual media platforms could enhance the effectiveness of malaria control initiatives [25–27] and promote a deeper understanding of the value of integrated healthcare practices.

Furthermore, THM providers operating outside integrated facilities proposed tax relief incentives from the central government to improve affordability for both patients and THM producers [38]. A robust financial support system could potentially increase access to quality malaria care at integrated hospitals, potentially leading to improved patient outcomes and reduced malaria prevalence [44, 45].

### Practice and research implications

The developed framework, “STRICT” (**S**tandardization of THM practice; **T**raining; **R**esearch; **I**ncrease awareness/integrated hospitals/inclusive NHIS; **C**onstant supply of approved herbal anti-malaria medications and **T**ax relief provision), offers guidance for delivering optimal patient care through integrated healthcare practices. This finding can inform policy modifications focused on malaria control and the implementation of integrated healthcare interventions. Potential policy changes include incorporating THM into medical school curriculum, establishing joint training programmes for healthcare providers, increasing the number of integrated hospitals, and expanding the NHIS to cover approved herbal anti-malarial medications. These strategies could enhance collaboration among healthcare providers, eliminate geographical and financial barriers to quality malaria care, and ultimately contribute to reduced malaria prevalence in Ghana, while strengthening the functionality of THM integration.

The framework further highlights key drivers for effective malaria control through integration. Healthcare managers can leverage participant recommendations on collaboration (training and research on THM intensified) and communication (the use of media, including radio, television, information centes) to raise awareness about the goal of integrated healthcare in reducing malaria burden. This would aid the delivery of quality health services for patients, especially those suffering from malaria and strengthen the efficiency of the integration intervention. By refining healthcare activities to reflect patient preferences through improved collaboration and communication, healthcare managers can strengthen the delivery of quality malaria services and the efficiency of the integrated healthcare intervention.

The need for further research in other malaria endemic countries with integrated healthcare systems is evident. Studies focusing on the experiences of key stakeholders, especially healthcare providers, could strengthen the evidence base and contribute to a clearer global understanding of how to effectively control malaria through integrated healthcare practices.

### Strengths and limitations

This study stands as the first qualitative exploration of stakeholder perspectives within the Ghanaian healthcare system to develop a framework for effective malaria control. The pluralistic approach ensured the inclusion of views from key participant groups (medical doctors, pharmacists and THM providers). Exploring the experiences of healthcare personnel directly involved in malaria care strengthens the research evidence.

However, limitations exist. The findings are specific to the Ghanaian context and may not be fully generalizable to other settings. Although eligible participants were recruited, the study could have been more comprehensive if the views of health service users (ultimate beneficiaries) and key decision makers were included. Lastly, participant responses could be subject to bias due to their affiliations and interest in integrated healthcare.

## Conclusion

This qualitative study explored the experiences and views of major stakeholders (medical doctors, pharmacists and THM providers) to develop the ‘STRICT’ framework, designed to enhance the functionality of the Ghanaian integrated healthcare system and promote effective malaria control. The research findings emphasize the importance of government policies that support THM training, research, standardization, and public awareness campaigns regarding integrated healthcare practices. Additionally, ensuring financial security for both THM providers and users diagnosed with malaria is crucial. These strategies can enhance collaboration between healthcare providers, increase utilization of THM services within integrated hospitals, and ultimately lead to improved patient experiences and positive health outcomes, including reduced malaria prevalence in Ghana.

## Data Availability

No datasets were generated or analysed during the current study.

## Abbreviations

ACT: Artemisinin-based Combination Therapy
AGA: Anglo Gold Ashanti
COREQ: Consolidated criteria for Reporting Qualitative studies
CSRPM: Centre for Scientific Research into Plant Medicine
FDA: Food and Drug Authority
GHS: Ghana Health Service
GSS: Ghana Statistical Service
ICF: International Classification of Functioning, disability and health
ITNs: Insecticide Treated Nets
KNUST: Kwame Nkrumah University of Science and Technology
MD: Medical Doctor
MOH: Ministry of Health
NHIS: National Health Insurance Scheme
NIHR: National Institute for Health and care Research
PM: Pharmacist
RSTMH: Royal Society of Tropical Medicine and Hygiene
THM: Traditional Herbal Medicine
TM: Traditional Medicine
UCC: University of Cape Coast
UDS: University of Development Studies
WHO: World Health Organization
